# Prognostic indicators associated with progresses of severe dengue

**DOI:** 10.1371/journal.pone.0262096

**Published:** 2022-01-05

**Authors:** Bùi Vũ Huy, Ngô Văn Toàn

**Affiliations:** 1 Department of Infectious Diseases, Hanoi Medical University, Hanoi, Vietnam; 2 Department of Pediatrics, National Hospital for Tropical Diseases, Hanoi, Vietnam; 3 Department of Environmental Health, Hanoi Medical University, Hanoi, Vietnam; Oregon State University, UNITED STATES

## Abstract

**Background:**

Dengue usually progress abnormally, especially in the critical phase. The main causes of death were shock, severe bleeding and organ failure. The aim of our study was to evaluate prognostic indicators of severe dengue according to the phases of the disease progression.

**Methods:**

A cross-sectional study was conducted from July to December 2017 at the National Hospital for Tropical Diseases and the Hospital for Tropical Diseases of Ho Chi Minh City. 326 patients, aged 6 years and over, including 99/326 patients with severe dengue and 227/326 patients with non-severe dengue, hospitalized in the first 3 days of illness, confirmed *Dengue* virus by the RT-PCR assay have been registered for the study. Clinical manifestations were monitored daily. The hematocrit, white blood cells, platelet, serum albumin, ALT, AST, bilirubin, prothrombin time (PT%, PTs), fibrinogen, aPTT, INR and creatinine were evaluated at two times: febrile phase and critical phase.

**Results:**

Independent factors associated with severe dengue were identified on multivariate logistic regression models. During the first 3 days of the disease, the prognostic indicators were platelet count ≤ 100 G/L (OR = 2.2; 95%CI: 1.2–3.9), or serum albumin < 35 g/L (OR = 3.3; 95%CI: 1.8–6.1). From day 4–6, the indicator were AST > 400 U/L (OR = 3.0; 95%CI: 1.1–7.9), ALT > 400 U/L (OR = 6.6; 95%CI: 1.7–24.6), albumin < 35 g/L (OR = 3.0; 95%CI: 1.5–5.9), and bilirubin total >17 μmol/L (OR = 4.6; 95%CI: 2.0–10.4).

**Conclusion:**

To predict the risk of patients with severe dengue, prognostic laboratory indicators should be indicated consistent with the progression of the disease. During the first 3 days of illness, prognostic indicators should be platelet count, or serum albumin. From the 4th - 6th day of illness, prognostic indicators should be AST, ALT, albumin, or bilirubin total.

## 1. Introduction

According to the announcement by WHO, the incidence of dengue has grown dramatically in recent decades throughout the world. There are an estimated 100 to 400 million dengue virus infections each year, and the regions of Southeast Asia, the Western Pacific and the Americas are the places most affected [1, 2]. In Vietnam, dengue disease has become a local epidemic and after a couple of years there is a serious outbreak of dengue [3].

Dengue disease usually progresses from 2 to 7 days, through three phases—febrile phase, critical phase and recovery phase. The main causes of death are serious clinical conditions, including shock, severe bleeding, and organ failure [1, 2]. According to WHO recommendations, most patients with dengue can be treated outpatient and screened for dengue cases with warning signs for hospitalization [2]. However, patients with dengue often have unusual progression, especially in the critical phase. In recent years, in addition to shock syndrome, serious conditions such as severe bleeding, impaired organ function in dengue patients have been reported with an increase [3–5]. The serious situation of liver failure, acute kidney injury (AKI) has also become a concern [6, 7] and affects treatment outcomes [5, 8–10]. In many cases, organ failure in dengue patients can only be detected based on the laboratory results [5, 9, 11]. Therefore, in addition to hematocrit and platelet indices [2], some recent studies have suggested the use of biomarkers such as AST, ALT [11–14], aPTT [12, 15, 16], and INR [10, 16] to predict severe or fatal dengue patients.

The aim of our study was to evaluate prognostic indicators of severe dengue according to the phases of the disease progression. The results of our study can provide additional indicators of prognostic value to avoid omitting severe dengue cases and limit the mortality rate.

## 2. Methods

### 2.1. Population

This study was conducted from July 1, 2017 to December 31, 2017. The patients were recruited from two specialized hospitals of infectious diseases in Vietnam, the National Hospital for Tropical Diseases (NHTD) in the North and the Hospital for Tropical Diseases of Ho Chi Minh City in the South. The inclusion criteria were as follows: (a) age ≥ 6 years, (b) clinical dengue, (c) confirmed *Dengue* virus (DENV) infection by real-time reverse-transcriptase polymerase chain reaction (rRT-PCR), and (d) hospitalized for the first 3 days of illness. Exclusion criteria were: a/ history of illness within 1 month, b/ any history of heart failure, liver failure, renal failure or bleeding disorder, c/being treated for viral hepatitis, because their clinical and laboratory findings may influence the results of the study. We also performed chest X ray and/or ultrasound examination to distinguish pleural effusion when patients admission.

#### 2.1.1. Study design

Cross sectional study. We used the formula for calculating the sample size for a cross-sectional study. Margin of error (d) was considered as 10% of the variance, expected prevalence (P) was 0.5, and Z_1-α/2_ = 1.96. Thus, the minimum sample size in each study group was 97 patients. In the progression of patients with dengue, the outcome was severe dengue and non-severe dengue. However, among patients with non-severe dengue, some experienced the warning signs recommended by WHO, but did not progress to severe dengue. Therefore, in this study, the non-severe dengue group included patients who experienced any warning signs and no warning signs, in equal numbers.

### 2.2. Conducted study

Study sites enrolled patients with suspected dengue based on clinical and NS1 test (+) [2], hospitalization during the first 3 days of illness. All patients were reviewed baseline characteristics and took 2ml of blood to determine DENV. These patients were followed for the first 10 days of illness. Research information of each patient was managed by individual records and codes, according to the pre-designed form.

Investigate indicators include [2, 4, 5, 7, 11] a/ clinical: Abdominal pain, persistent vomiting, mucosal bleed, liver enlargement > 2 cm; b/ laboratory: Hematocrit, white blood cells (WBC), platelet count, serum albumin, alanine aminotransferase (ALT), aspartate aminotransferase (AST), bilirubin total (bilirubin TT), prothrombin time (PT%, PTs), fibrinogen, activated partial thromboplastin time (aPTT), international normalized ratio (INR) and creatinine. Clinical signs and symptoms were monitored daily. Laboratory indicators were evaluated at 2 times: febrile phase (days 1–3), critical phase (days 4–6). Electrocardiogram or ultrasound of the abdomen, pleura, and pericardium were performed when abnormalities were suspected. At the end of clinical follow-up, based on clinical progress, the patient was clinically classified as severe dengue and dengue without warning signs/any warning signs. The patient list (including identification code, clinical classification) and their blood samples for DENV identification were sent to the NHTD virology laboratory. The virology laboratory performs the determination of DENV according to the clinical classification. Samples (patients) with PCR (+) results were collected sequentially over time and ensured a minimum of 97 patients for each clinical classification of severe dengue and dengue without warning signs and with any warning sign.

### 2.3. Definitions

All clinical classification criteria and treatment regimens were performed according to the 2009 WHO guidelines [2]. Severe dengue was defined by any of the following criteria: a) there was evidence of shock or pleural/peritoneal fluid retention causing respiratory failure; b) severe bleeding that required intervention, or; c) severe organ impairment, such as acute liver failure (AST, ALT ≥ 1000 U/L [2]), AKI (serum creatinine level increased ≥ 0.3 mg/dL within 48 hours or elevated ≥ 1.5-fold from baseline or within 07 days [17]), encephalopathy (if there is evidence of convulsions or consciousness disturbances), or evidence of myocarditis, heart failure.

In this study, peripheral blood parameters were performed on Beckman coulter DXH 600 system. Liver and kidney function was performed on AU 480 Backman Counter system. Coagulation parameters were performed on ACL TOP 300. Using SD Bioline’s NS1 rapid test, the sensitivity and specificity were 92.4% and 98.4%. For the viral diagnosis, in brief, the viral RNA was extracted using Viral RNA mini kit (Qiagen company, Cat. No. 52904, Chatsworth, CA, USA), DENV (1–4) primers and probes Sigma–Proligo, according to the manufacturer’s instructions. The cDNA was reverse transcription from RNA of DENV and thereafter used as a template for dengue viral serotypes by real-time reverse transcriptase-polymerase chain reaction (RT-PCR) using Light Cycler 480 (Roche).

### 2.4. Data analysis

All data were analyzed by the SPSS software (version 16.0). Categorical variables were expressed as frequencies and percentages. To determine the prognostic indicators of severe dengue, patients were divided into 2 groups: severe dengue and non-severe dengue (without warning signs/with any warning signs). We conducted analysis the study indicators according to the phases of the disease, the febrile phase (days 1–3 of the illness) and the critical phase (days 4–6 of the illness). Univariate logistic regression analysis was performed with each potential factor as an independent variable and the presence or absence of severe dengue as the dependent variable. Any variable with a p-value ≤ 0.05 was considered potentially significant and was further analyzed in a stepwise multivariate logistic regression analysis to determining significant independent factors associated with severe dengue. The values of laboratory indices at upper (or lower) thresholds, or optimal cut-off values were also determined for each phase of the disease in the regression models.

#### 2.4.1. Ethical considerations

The study design was approved by the Ethics Committee of the NHTD (document no: 23/HĐĐĐ-NĐTƯ). Written informed consent was obtained from all patients, or the patient’s parent/guardians if the patient was under 18 years of age, before participation in the study.

## 3. Results

### 3.1. Patients characteristics

A total of 326 patients identified with DENV infection based on PCR results were enrolled in this study. The clinical classification of 326 patients were 99/326 (30.4%) severe dengue, and 227 (69.6%) non-severe dengue including 117/326 (35.9%) without warning signs, 110/326 (33.7%) with any warning signs. The baseline, clinical and laboratory characteristics of the study patients were presented in Table 1. Patients in the study were distributed in all ages from 06 years to 91 years, in which men accounted for 50.9%. The underlying diseases included: inflammation/ulceration of stomach (9 patients), diabetes (5 patients), cancer (2 patients), chronic lung disease (1 patient), and other diseases such as beta thalassemia, luput. Among the patients with underlying disease who were enrolled in the study, there were also 3 patients with a history of viral hepatitis, but no evidence of active hepatitis and no indication for viral hepatitis treatment; Six had a history of cardiovascular/hypertension, but all showed no signs of heart failure.

**Table 1:** Baseline, clinical and laboratories characteristics of study patients.

Variable	Total (N = 326)	Severe dengue (N = 99)	Non severe dengue (N = 227)
	n (%)	n (%)	n (%)
**Baseline characteristics**			
Gender			
• Male	166 (50,9)	51 (51.5)	115 (50.7)
• Female	160 (49,1)	48 (48.5)	112 (49.3)
Age			
• 6–10 years	32 (9.8)	10 (10.1)	9.7)
• 11–20 years	112 (34.4)	26 (26.3)	37.9)
• 21–30 years	89 (27.3)	37 (37.4)	22.9)
• 31–40 years	44 (13.5)	8 (8.1)	15.9)
• 41–50 years	32 (9.8)	12 (12.1)	8.8)
• ≥ 51 years	17 (5.2)	6 (6.1)	11 (4.8)
Underlying disease			
• Yes	40 (12.3)	11 (11.1)	12.8)
• No	286 (87.7)	88 (88.9)	198 (87.2)
**Clinical manifestations**			
Fever	326 (100.0)	99 (100.0)	227 (100.0)
Skin rash	251 (77.0)	65 (65.7)	186 (81.9)
Myalgia	215 (66.0)	60 (60.6)	155 (68.3)
Haemorrhagic manifestations	190 (58.3)	56 (56.6)	9.0)
• Petechiae	106 (32.5)	32 (32.3)	32.6)
• Mucosal bleeding	149 (45.7)	41 (41.4)	108 (47.6)
Vomiting and /or nausea	159 (48.8)	39 (39.4)	120 (52.9)
Abdominal pain	113 (34.7)	36 (36.4)	77 (33.9)
Loose stools	54 (16.6)	14 (14.1)	40 (17.6)
Hepatomegaly	13 (4.0)	7 (7.1)	6 (2.6)
**Peripheral blood**			
• Hematocrit > 0.4 l/L	208 (63.8)	78 (78.8)	9.9)
• WBC < 5 G/L	257 (78.8)	72 (72.7)	1.5)
• Platelets ≤ 100 G/L	213 (65.3)	79 (79.8)	134 (59.0)
**Biochemical parameters**			
Creatinin > 120 μmol/L	30 (9.2)	30 (30.3)	0 (0.0)
Liver enzymes			
• AST > 40 U/L	269 (82.5)	87 (87.9)	182 (80.2)
• ALT > 40 U/L	210 (64.4)	81 (81.8)	129 (56.8)
Albumin < 35 g/L	57 (17.5)	32 (32.3)	25/226 (11.1)
Bilirubin TT > 17 μmol/L	39 (12.0)	27 (27.3)	12 (5.3)
Glucose			
• ≤ 3.9 mmol/L	6 (1.9)	3/98 (3.1)	3 (1.3)
• ≥ 6.4 mmol/L	2 (0.6)	0 (0.0)	2/225 (0.9)
**Coagulation parameters**			
• PT < 70%	26 (8.0)	13 (13.1)	13 (5.7)
• PTs > 13 (s)	90 (27.6)	40 (40.4)	50 (22.0)
• Fibrinogen < 2 g/L	90 (27.9)	42 (42.4)	48 (21.1)
• aPTT > 40 (s)	163 (50.0)	49 (49.5)	114 (50.2)
• INR > 1.25 (s)	28 (8.6)	11 (11.1)	17 (7.5)
**DENV type**			
• DEN-1	316 (96.9)	94 (94.9)	222 (97.8)
• DEN-2	10 (3.1)	5 (5.1)	5 (2.2)

The main clinical manifestations were recorded during follow-up, including fever (100%), skin rash (77.0%), myalgia (66.0%), bleeding (58.3%). Less common manifestations were vomiting, abdominal pain, loose stools, hepatomegaly. Laboratory parameters for hematology, biochemistry and coagulation were abnormal with different rates. In addition to an increase in the hematocrits, a decrease in platelet and white blood cell counts, there was an above-threshold increase in serum creatinine, ALT, AST, bilirubin total and a decrease in serum albumin, as well as disturbances in coagulation parameters.

Among 99 patients classified as severe dengue included shock/severe effusion (40 patients), organ failure (39 patients) and severe bleeding (20 patients).

### 3.2. The prognostic factors associated with disease progression

In the process of evolution of patients with dengue, the observed study indicators have changed with a tendency to worsen, in both severe and non-severe dengue groups. Clinically, the manifestations of vomiting, abdominal pain, mucosal bleeding were observed from the febrile phase, but hepatomegaly was detected only in the critical phase (Tables 2 and 3). The evaluated laboratory indicators, including those evaluated based on cut-off points, as well as those evaluated against the threshold (above or below the threshold) all changed in the negative direction. Specifically, the cut-off point of the platelet count if in the febrile phase was 100 G/L, then in the dangerous phase was 50 G/L. Similarly, cut-off point of hematocrit increased from 0.4 l/L to 0.42 l/L and AST/ALT increased from 200 U/L to 400 U/L from febrile phase to critical phase. Based on clinical and laboratory results for each phase of disease progression, we have included univariate and multivariate analysis models to determine prognostic indicators of severe dengue.

**Table 2:** Regression analysis of factors associated with severe dengue based on clinical and laboratory parameters, during the first three days of illness.

Indicators	Univariate analysis		Multivariate analysis	
	P	OR (95% CI)	P	OR (95% CI)
Vomit	> 0.05	0.9(0.4–1.9)		
Abdominal pain	> 0.05	0.8(0.3–2.0)		
Mucosal bleeding	> 0.05	0.1(0.02–1.1)		
Hematocrit > 0.4 l/L	< 0.05	1.7(1.1–2.8)	> 0.05	1.5 (0.9–2.6)
WBC < 5 G/L	> 0.05	0.9(0.5–1.4)		
Platelet ≤ 100 G/L	< 0.01	2.5(1.5–4.4)	< 0.05	2.2 (1.2–3.9)
AST > 200 U/L	< 0.01	4.6 (1.6–12.8)	> 0.05	1.9 (0.4–9.6)
ALT > 200 U/L	< 0.01	7.6 (2.4–24.3)	> 0.05	4.4 (0.9–20.8)
Albumin < 35 g/L	< 0.01	3.1 (1.8–5.5)	< 0.01	3.3 (1.8–6.1)
Bilirubin TT > 17 μmol/L	> 0.05	1.8 (0.8–4.3)		
Creatinin > 120 μmol/L	Not significant	Not available		
Glucose ≤ 3.9 mmol/L	Not significant	Not available		
PT < 70%	> 0.05	0.9(0.5–1.5)		
PTs > 13 (s)	> 0.05	1.2(0.8–2.0)		
Fibrinogen < 2 g/L	> 0.05	1.8(0.9–3.4)		
aPTT > 40 (s)	< 0.01	2.2(1.4–3.7)	> 0.05	1.7 (0.9–2.9)
INR > 1.25 (s)	> 0.05	1.1(0.6–1.8)		

**Table 3:** Regression analysis of factors associated with severe dengue based on clinical and laboratory parameters, from day 4–6 of the illness.

Indicators	Univariate analysis		Multivariate analysis	
	P	OR (95% CI)	P	OR (95% CI)
Vomit	> 0.05	0.9(0.5–1.6)		
Abdominal pain	> 0.05	1.5(0.9–2.5)		
Mucosal bleeding	> 0.05	0.8(0.5–1.2)		
Hepatomegaly	> 0.05	1.3(0.7–2.3)		
Hematocrit > 0.42 l/L	> 0.05	1.5(0.9–2.4)		
WBC < 5 G/L	> 0.05	0.6(0.3–1.0)		
Platelet ≤ 50 G/L	< 0.01	2.1 (1.3–3.4)	> 0.05	1.5 (0.8–2.6)
AST > 400 U/L	< 0.01	8.2(3.9–16.8)	< 0.05	3.0 (1.1–7.9)
ALT > 400 U/L	< 0.01	17.8(5.9–53.1)	< 0.01	6.6 (1.7–24.6)
Albumin < 35 g/L	< 0.01	3.8(2.1–6.9)	< 0.01	3.0 (1.5–5.9)
Bilirubin TT > 17 μmol/L	< 0.01	6.7(3.2–13.9)	< 0.01	4.6 (2.0–10.4)
Creatinin > 120 μmol/L	Not significant	Not available		
Glucose ≤ 3.9 mmol/L	> 0.05	2.3 (0.5–11.8)		
PT < 70%	< 0.05	2.5(1.1–5.6)	> 0.05	1.9 (0.7–4.9)
PTs > 13 (s)	>0.05	0.4(0.03–7.0)		
Fibrinogen < 2 g/L	< 0.01	2.7(1.6–4.5)	> 0.05	1.8 (0.9–3.3)
aPTT > 40 (s)	> 0.05	0.9(0.6–1.5)		
INR > 1.25 (s)	> 0.05	1.6(0.7–3.6)		

In the first three days of the disease, the results of univariate logistic regression analysis showed that the indicators related to severe dengue were hematocrit > 0.4 l/L, platelet ≤ 100 G/L, AST/ALT > 200 U/L, serum albumin < 35 g/L. Multivariate analysis results showed that platelet ≤ 100 G/L and albumin < 35 g/L were independent prognostic indicators of severe dengue.

From day 4–6 of the disease, the results of univariate logistic regression analysis showed that the indicators related to the prognosis of severe dengue were platelets ≤ 50 G/L, AST/ALT > 400 U/L, serum albumin < 35 g/L, bilirubin TT > 17 μmol/L, PT <70% and fibrinogen < 2 g/L. The results of multivariate regression analysis showed that enzyme AST/ALT > 400 UI/L, serum albumin < 35 g/L and bilirubin TT > 17 μmol/L were independent prognostic indicators of severe dengue.

## 4. Discussion

### 4.1. Clinical characteristics of studied patients

In this study, study patients were diagnosed with dengue based on PCR assays, of which DEN 1 accounted for 96.9% and DEN-2 accounted for 3.1% (Table 1). In order to determine prognostic indicators of severe dengue, the patients were selected for the purposes of the study, so we did not focus on analyzing the clinical and laboratory characteristics. However, patients who participated in our research demonstrated the characteristics of patients with dengue in the current period. In terms of demographics, patients distributed in all ages, from 06 years old (age included in the study) to 91 years old, mainly in the 11–40 years old group, men sex accounted for 50.9%. Studies in dengue patients also recorded no difference in sex in patients infected with DENV, and the age group from 11 to 40 years was most common [3, 18, 19]. The underlying diseases of the patients participating in our study have also been recognized as common diseases in dengue patients [3, 19]. Clinically, common manifestations including fever, skin rash, myalgia and hemorrhage, as well as less common manifestations such as vomiting, abdominal pain, loose stools, hepatomegaly have been reported in dengue patient [2]. In peripheral blood, an increase in hematocrit and a decrease in platelet counts below ≤ 100 G/L were also observed in this study. A rising haematocrit of 10% above baseline is an early objective indicator of plasma leakage [4].

The study results also showed that although the proportion of patients with severe dengue was only 30.4% of the total number of patients enrolled in the study, however, the proportion of patients with hematocrit > 40% was 63.8%. Furthermore, the function of many organs also has abnormal changes in dengue patients. The rate of abnormal increase in serum creatinine was 9.2%, serum albumin < 35 g/L was 17.5%, enzymes ALT and AST > 40 U/L ranged from 64.4% - 82.5%, bilirubin TT > 17 mmol/L was 12%. There was also a disorder of the 5 coagulation parameters ranged from 8.0% to 50% (Table 1).

In patients with dengue, abnormal manifestations have been reported [2, 4]. AKI in dengue patients may recover on its own, but may also require kidney replacement therapy [7, 20]. Liver function damage is relatively common in dengue patients and can lead to acute liver failure [6, 10]. Disorders of coagulation parameters such as APTT, PT, fibrinogen are common in patients with severe dengue [12], leading to severe bleeding, especially in prolonged shock [2, 4].

Therefore, patients in our study were not only identified DENV by PCR, but also had the baseline, clinical and peripheral blood characteristics of dengue disease in the current period.

### 4.2. Prognostic factors for severe dengue according to the phases of disease progression

In this study, we evaluated parameters, including peripheral blood cells, liver function, creatinine, and 5 baseline coagulation factors that were commonly performed in clinical practice. The results of our study showed that a decreased serum albumin < 35 g/L was a predictive indicator for severe dengue in both phases of the disease (Tables 2 and 3). Status decreased serum albumin has also been recognized indirectly reflect the increase in vascular permeability and lead to plasma leakage and shock in dengue disease [2, 4]. It was noteworthy that the serum albumin as prognostic indicator for severe dengue from early phase of the disease. The indicators of AST and ALT have been suggested in the prognosis of severe dengue by many studies, however the recommendations were also very different [11–13, 21]. According to a retrospective study result, on day 3 of the disease, if AST ≥ 203 U/L or ALT ≥ 55 U/L was proposed as prognostic indicator of early mortality [12]. Another retrospective study suggested cut-off values of AST and ALT with two choices of 402 U/L and 653 U/L for severe dengue prognosis, nevertheless this study did not suggest timing of evaluation [11]. In our study, in the febrile phase, although the univariate analysis showed that cut-off values of ALT/AST > 200 U/L were prognostic values, but in the multivariate analysis there were no prognostic values for severe dengue. Similarly, the cut-off values of ALT/AST > 400 U/L only have prognostic value severe dengue in the critical phase. Therefore, the parameters ALT/AST were prognosis indicators for severe dengue, nevertheless this indicators needs to be indicated at the right time and be interested to threshold scores with prognostic value (Table 3). Peripheral blood parameters such as hematocrit > 40% or platelet count <50 G/L have been suggested as indicators for the prognosis of severe dengue [22]. However, the results of our study showed that the hematocrit parameter was no value in the prognosis of severe dengue. It is possible that early infusion according to WHO guidelines has compensated for the amount of leak fluid. Platelet counts < 50 G/L were also observed in patients with non-severe dengue and severe dengue [6, 23]. Severe bleeding may not occur even if the platelet count drops by 10 G/L in patients with non-severe dengue [4]. Thrombocytopenia combined with coagulation abnormalities were thought to increase the risk of bleeding in patients with profound shock [2]. However, if during the febrile phase, the platelet count dropped by < 100 G/L, which would be a prognostic indicator of severe dengue (Table 2). The decreased number of platelets in the peripheral blood is associated to a number of mechanisms, such as the suppression of bone marrow by DENV, by antiplatelet antibodies or a mitochondrial disorder. In other words, it is due to the virulence of the DENV [24]. This can lead to a severe dengue illness.

Our results study also showed that during the critical phase, an increase in bilirubin > 17 μmol/L was a prognostic indicator of severe dengue (Table 3). An increase in bilirubin reflected the hepatic dysfunction in DENV infection. This was either a result of direct viral toxicity or compromised immune response to the virus [25]. Although the bilirubin parameter was rarely suggested, however some studies have also recognized that bilirubin may be associated with severe dengue prognosis [10].

As recommended by WHO, warning signs of severe dengue are lethargy or irritability, persistent vomiting, abdominal pain or tenderness, enlarged liver, mucosal bleed, low urination, and increase in hematocrit concurrent with rapid decrease in platelet count [2, 4]. The Warning signs were also observed in our study. However some signs such as lethargy, vomiting and abdominal pain improved rapidly with infusion interventions according to WHO guidelines. Despite this, status of shock, or severe bleeding or organ failure still occurred in some patients. In our opinion, the above signs were also subjective, especially in the elderly or in young children. In contrast, signs such as lethargy, irritation, vomiting were not observed in patients with organ failure such as AKI or hepatic impairment. Limitations of the study: First, this was a multicenter study, so the bias cannot be avoided. To limit errors, we have designed study records form and conducted pre-research training in clinical and laboratory techniques. Second, the study was evaluated only at ages 6 and over, not at all ages. Third, we did not have agreement on the criteria for an increase in hematocrit concurrently with the rapidly decreasing platelet count for implementation in this study. Therefore this standard has not been evaluated. However, the results of this study will provide information’s that should be considered in the prognosis of severe dengue.

## 5. Conclusion

To prognosis patients with severe dengue should be evaluated according to the phase of the disease. In the first 3 days of the disease, the recommended indicator should be platelet count ≤ 100 G/L (OR = 2.2; 95%CI: 1.2–3.9) or serum albumin <35 g/L (OR = 3.3; 95%CI: 1.8–6.1). From day 4–6 of the disease, the recommended indicators should be AST > 400 U/L (OR = 3.0; 95%CI: 1.1–7.9), ALT > 400 U/L (OR = 6.6; 95%CI: 1.7–24.6), albumin < 35 g/L (OR = 3.0; 95%CI: 1.5–5.9) và bilirubin TT > 17 μmol/L (OR = 4.6; 95%CI: 2.0–10.4).

## Supporting information

S1 Table The data supporting the analysis of the results of Table 2. (DOCX)

S2 Table The data supporting the analysis of the results of Table 3. (DOCX)
