# Supplementary material for: Prognostic indicators associated with progresses of severe dengue
Source: PLoS One. 2022 Jan 5;17(1):e0262096. doi: 10.1371/journal.pone.0262096 (PMC8730386; doi:10.1371/journal.pone.0262096)
Supplement: S1 Table — (DOCX) [file pone.0262096.s001.docx]

The data supporting the analysis of the results of Table 2

Table 2: Bivariate an multivariate analysis of factors associated with severe dengue based on clinical and laboratory parameters, during the first three days of illness

| **Indicators** | **No severe dengue**  **n =227**  **(n,%)** | **Severe dengue**  **n =99**  **(n,%)** | **P^*^** | **OR (95% CI)** |
| --- | --- | --- | --- | --- |
| Vomit | 25 (11,0) | 10 (10,1) | > 0.05 | 0.9(0.4-1.9) |
| Abdominal pain | 19 (8,4) | 7 (7,1) | > 0.05 | 0.8(0.3-2.0) |
| Mucosal bleeding | 15 (6.6) | 1 (1.0) | > 0.05 | 0.1(0.02-1.1) |
| *Hematocrit > 0,4 L/L* | *88 (37,8)* | *52 (52,5)* | **< 0.05** | **1.7(1.1-2.8)** |
| WBC < 5 G/L | 133 (58,6) | 55 (55,6) | > 0.05 | 0.9(0.5-1.4) |
| Platelet *≤ 100 G/L* | *40 (17,6)* | *35 (35,4)* | ***< 0.01*** | ***2.5(1.5-4.4)*** |
| Liver enzymes (U/L) |  |  |  |  |
| *- AST > 200* | *6 (2,7)* | *11 (11,1)* | ***< 0,01*** | ***4,6 (1,6-12,8)*** |
| *- ALT > 200* | *4 (1,8)* | *12 (12,1)* | ***< 0,01*** | ***7,6 (2,4-24,3)*** |
| *Albumin < 35 g/L* | *37 (16,3)* | *29 (29,3)* | ***< 0,01*** | ***3.1 (1.8-5.5)*** |
| Bilirubin TP >17(μmol/L) | 13 (5,8) | 10 (10,1) | > 0.05 | 1.8 (0.8-4.3) |
| Creatinin >120 μmol/L | 0 (0,0) | 30 (30,3) | - | N/A |
| Glucose ≤ 3,9 mmol/L | 0(0,0) | 1 (1,0) | - | N/A |
| PT < 70 % | 72 (31,9) | 30 (30,3) | > 0.05 | 0.9(0.5-1.5) |
| PTs > 13 s | 134 (59,3) | 64 (64,6) | > 0.05 | 1.2(0.8-2.0) |
| Fibrinogen < 2 g/L | 26 (11,8) | 19 (19,2) | > 0.05 | 1.8(0.9-3.4) |
| *APTT > 40 s* | *62 (27,4)* | *46 (46,5)* | **< 0.01** | **2.2(1.4-3.7)** |
| INR > 1,25 | 62 (27,3) | 29 (29,3) | > 0.05 | 1.1(0.6-1.8) |

**Regression Multivariate analysis of factors associated with severe dengue based on clinical and laboratory parameters, during the first three days of illness**

| **Variables in the Equation** | | | | | | | | | |
| --- | --- | --- | --- | --- | --- | --- | --- | --- | --- |
|  |  | B | S.E. | Wald | df | Sig. | Exp(B) | 95.0% C.I.for EXP(B) | |
|  |  |  |  |  |  |  |  | Lower | Upper |
| Step 1^a^ | PL.hct13 | .427 | .270 | 2.509 | 1 | .113 | 1.533 | .904 | 2.601 |
|  | PL.PlT13 | .775 | .306 | 6.426 | 1 | .011 | 2.171 | 1.192 | 3.952 |
|  | PL.ast13 | .632 | .831 | .579 | 1 | .447 | 1.882 | .369 | 9.598 |
|  | PL.alt13 | 1.478 | .794 | 3.466 | 1 | .063 | 4.385 | .925 | 20.789 |
|  | PL.albumin13 | 1.196 | .316 | 14.315 | 1 | .000 | 3.306 | 1.779 | 6.142 |
|  | PL.aptt13 | .520 | .282 | 3.393 | 1 | .065 | 1.682 | .967 | 2.924 |
|  | Constant | -6.802 | 1.050 | 41.942 | 1 | .000 | .001 |  |  |
| 1. Variable(s) entered on step 1: PL.hct13, PL.TC13, PL.ast13, PL.alt13, PL.albumin13, PL.aptt13. | | | | | | | | |  |

| **Indicators** | **No severe dengue**  **n =227**  **(n,%)** | **Severe dengue**  **n =99**  **(n,%)** | **Crude OR (95% CI)** | **Adjusted OR (95% CI)** |
| --- | --- | --- | --- | --- |
| Vomit | 25 (11,0) | 10 (10,1) | 0.9(0.4-1.9) |  |
| Abdominal pain | 19 (8,4) | 7 (7,1) | 0.8(0.3-2.0) |  |
| Mucosal bleeding | 15 (6.6) | 1 (1.0) | 0.1(0.02-1.1) |  |
| Hematocrit > 0,4 L/L | *88 (37,8)* | *52 (52,5)* | **1.7 (1.1-2.8)** | 1.5 (0.9 – 2.6) |
| WBC < 5 G/L | 133 (58,6) | 55 (55,6) | 0.9(0.5-1.4) |  |
| Platelet *≤ 100 G/L* | *40 (17,6)* | *35 (35,4)* | ***2.5(1.5-4.4)*** | 2.2 (1.2 – 3.9) |
| AST > 200 | *6 (2,7)* | *11 (11,1)* | ***4,6 (1,6-12,8)*** | 1.9 (0.4 – 9.6) |
| ALT > 200 | *4 (1,8)* | *12 (12,1)* | ***7,6 (2,4-24,3)*** | 4.4 (0.9 – 20.8) |
| *Albumin < 35 g/L* | *37 (16,3)* | *29 (29,3)* | ***3.1 (1.8-5.5)*** | 3.3 (1.8 – 6.1) |
| Bilirubin TP >17(μmol/L) | 13 (5,8) | 10 (10,1) | 1.8 (0.8-4.3) |  |
| Creatinin >120 μmol/L | 0 (0,0) | 30 (30,3) | N/A |  |
| Glucose ≤ 3,9 mmol/L | 0(0,0) | 1 (1,0) | N/A |  |
| PT < 70 % | 72 (31,9) | 30 (30,3) | 0.9(0.5-1.5) |  |
| PTs > 13 s | 134 (59,3) | 64 (64,6) | 1.2(0.8-2.0) |  |
| Fibrinogen < 2 g/L | 26 (11,8) | 19 (19,2) | 1.8(0.9-3.4) |  |
| APTT > 40 s | *62 (27,4)* | *46 (46,5)* | **2.2(1.4-3.7)** | 1.7 (0.9 – 2.9) |
| INR > 1,25 | 62 (27,3) | 29 (29,3) | 1.1(0.6-1.8) |  |
