# Supplementary material for: Prognostic indicators associated with progresses of severe dengue
Source: PLoS One. 2022 Jan 5;17(1):e0262096. doi: 10.1371/journal.pone.0262096 (PMC8730386; doi:10.1371/journal.pone.0262096)
Supplement: S2 Table — (DOCX) [file pone.0262096.s002.docx]

The data supporting the analysis of the results of Table 3

***Table 3:* Regression univariate analysis of factors associated with severe dengue based on clinical and laboratory parameters, from day 4 – 6 of illness**

| **Indicators** | **No severe dengue**  **(n,%)** | **Severe dengue**  **(n,%)** | **P^*^** | **OR(95% CI)** |
| --- | --- | --- | --- | --- |
| Vomit | 47 (20,7) | 19 (19,2) | > 0.05 | 0.9(0.5-1.6) |
| Abdominal pain | 58 (25,6) | 34 (34,3) | > 0.05 | 1.5(0.9-2.5) |
| Mucosal bleeding | 108 (47,6) | 41 (41,4) | > 0.05 | 0.8(0.5 – 1.2) |
| Hepatomegaly | 6 (2,6) | 7 (7,1) | > 0.05 | 1.3(0.7- 2.3) |
| Hematocrit > 0,42 l/l | 100 (44,1) | 54 (54,5) | > 0.05 | 1.5(0.9-2.4) |
| WBC < 5 G/L | 185 (81,5) | 72 (72,7) | > 0.05 | 0.6(0.3-1.0) |
| Platelet *≤ 50 G/L* | *72 (31,7)* | *49 (49,5)* | ***< 0.01*** | ***2.1 (1.3-3.4)*** |
| Creatinin >120 μmol/L | 0 (0,0) | 30 (30,3) | - | N/A |
| Enzym gan (U/L) |  |  |  |  |
| *- AST > 400* | *12 (5,3)* | *31 (31,3)* | ***< 0.01*** | ***8.2(3.9-16.8)*** |
| *- ALT > 400* | *4 (1,8)* | *24 (24,2)* | ***< 0.01*** | ***17.8(5.9-53.1)*** |
| *Albumin < 35 g/L* | *25 (11,1)* | *32 (32,2)* | ***< 0.01*** | ***3.8(2.1-6.9)*** |
| *Bilirubin TT > 17 (μmol/L)* | *12 (5,3)* | *27 (27,3)* | ***< 0.01*** | ***6.7(3.2-13.9)*** |
| Glucose ≤ 3.9 mmol/L | 3 (1,3) | 2 (2,0) | > 0.05 | 2.3 (0.5-11.8) |
| *PT < 70 %* | *13 (5,7)* | *13 (13,1)* | ***< 0.05*** | ***2.5(1.1-5.6)*** |
| *PTs > 13 (s)* | *50 (22,0)* | *40 (40,4)* | >0.05 | 0.4(0.03-7.0) |
| *Fibrinogen < 2 g/l* | *48 (21,4)* | *42 (42,4)* | ***< 0.01*** | ***2.7(1.6-4.5)*** |
| APTT > 40 (s) | 114 (50,2) | 49 (49,5) | > 0.05 | 0.9(0.6-1.5) |
| INR > 1,25 (s) | 16 (7,0) | 11 (11,1) | > 0.05 | 1.6(0.7-3.6) |

******* *Univariate logistic regression analysis*

**Regression Multivariate analysis of factors associated with severe dengue based on clinical and laboratory parameters, from day 4-6 of the illness**

| **Variables in the Equation** | | | | | | | | | |
| --- | --- | --- | --- | --- | --- | --- | --- | --- | --- |
|  |  | B | S.E. | Wald | df | Sig. | Exp(B) | 95.0% C.I.for EXP(B) | |
|  |  |  |  |  |  |  |  | Lower | Upper |
| Step 1^a^ | PL.PLT 2.6 | .400 | .294 | 1.857 | 1 | .173 | 1.492 | .839 | 2.652 |
|  | PL.ast6 | 1.123 | .492 | 5.206 | 1 | .023 | 3.075 | 1.172 | 8.073 |
|  | PL.alt6 | 1.872 | .674 | 7.716 | 1 | .005 | 6.500 | 1.735 | 24.347 |
|  | PL.albu6 | 1.110 | .345 | 10.341 | 1 | .001 | 3.036 | 1.543 | 5.973 |
|  | PL.BilTT6 | 1.538 | .416 | 13.673 | 1 | .000 | 4.656 | 2.060 | 10.521 |
|  | PL.PT6 | .591 | .506 | 1.367 | 1 | .242 | 1.806 | .671 | 4.864 |
|  | PL.PTs6 | -.623 | 1.517 | .169 | 1 | .681 | .536 | .027 | 10.499 |
|  | PL.fibrino6 | .585 | .312 | 3.522 | 1 | .061 | 1.795 | .974 | 3.306 |
|  | Constant | -7.973 | 3.348 | 5.671 | 1 | .017 | .000 |  |  |
| a. Variable(s) entered on step 1: PL.tc2.6, PL.ast6, PL.alt6, PL.albu6, PL.BilTP6, PL.PT6, PL.PTs6, PL.fibrino6. | | | | | | | | | |

| **Indicators** | **No severe dengue**  **(n,%)** | **Severe dengue**  **(n,%)** | **Crude OR OR(95% CI)** | **Adjusted OR (95% CI)** |
| --- | --- | --- | --- | --- |
| Vomit | 47 (20,7) | 19 (19,2) | 0.9(0.5-1.6) |  |
| Abdominal pain | 58 (25,6) | 34 (34,3) | 1.5(0.9-2.5) |  |
| Mucosal bleeding | 108 (47,6) | 41 (41,4) | 0.8(0.5 – 1.2) |  |
| Hepatomegaly | 6 (2,6) | 7 (7,1) | 1.3(0.7- 2.3) |  |
| Hematocrit > 0,42 l/l | 100 (44,1) | 54 (54,5) | 1.5(0.9-2.4) |  |
| WBC < 5 G/L | 185 (81,5) | 72 (72,7) | 0.6(0.3-1.0) |  |
| Platelet *≤ 50 G/L* | *72 (31,7)* | *49 (49,5)* | ***2.1 (1.3-3.4)*** | 1.5 (0.8 – 2.6) |
| *- AST > 400* | *12 (5,3)* | *31 (31,3)* | ***8.2(3.9-16.8)*** | 3.0 (1.1 – 7.9) |
| *- ALT > 400* | *4 (1,8)* | *24 (24,2)* | ***17.8(5.9-53.1)*** | 6.6 (1.7 – 24.6) |
| *Albumin < 35 g/L* | *25 (11,1)* | *32 (32,2)* | ***3.8(2.1-6.9)*** | 3.0 (1.5 – 5.9) |
| *Bilirubin TT > 17 (μmol/L)* | *12 (5,3)* | *27 (27,3)* | ***6.7(3.2-13.9)*** | 4.6 (2.0 – 10.4) |
| Creatinin >120 μmol/L | 0 (0,0) | 30 (30,3) | N/A |  |
| Glucose ≤ 3.9 mmol/L | 3 (1,3) | 2 (2,0) | 2.3 (0.5-11.8) |  |
| *PT < 70 %* | *13 (5,7)* | *13 (13,1)* | ***2.5(1.1-5.6)*** | 1.9 (0.7-4.9) |
| *PTs > 13 (s)* | *50 (22,0)* | *40 (40,4)* | 0.4(0.03-7.0) |  |
| *Fibrinogen < 2 g/l* | *48 (21,4)* | *42 (42,4)* | ***2.7(1.6-4.5)*** | 1.8 (0.9 – 3.3) |
| APTT > 40 (s) | 114 (50,2) | 49 (49,5) | 0.9(0.6-1.5) |  |
| INR > 1,25 (s) | 16 (7,0) | 11 (11,1) | 1.6(0.7-3.6) |  |
